# Impact of low eGFR on the immune response against COVID-19

**DOI:** 10.1007/s40620-022-01374-1

**Published:** 2022-07-02

**Authors:** Arturo Blazquez-Navarro, Lisa Mittmann, Constantin Joachim Thieme, Moritz Anft, Krystallenia Paniskaki, Adrian Doevelaar, Felix Sebastian Seibert, Bodo Hoelzer, Margarete Justine Konik, Marc Moritz Berger, Thorsten Brenner, Clemens Tempfer, Carsten Watzl, Toni Luise Meister, Stephanie Pfaender, Eike Steinmann, Sebastian Dolff, Ulf Dittmer, Oliver Witzke, Ulrik Stervbo, Toralf Roch, Michal Or-Guil, Timm Henning Westhoff, Nina Babel

**Affiliations:** 1 Center for Translational Medicine and Immune Diagnostics Laboratory, Medical Department I, Ruhr-University Bochum, Marien Hospital Herne, University Hospital of the Ruhr-University Bochum, Herne, North Rhine-Westphalia Germany; 2 BIH Center for Regenerative Therapies, Charité–Universitätsmedizin Berlin, Corporate Member of Freie Universität Berlin, Humboldt-Universität Zu Berlin, and Berlin Institute of Health, Berlin, Germany; 3 Department of Infectious Diseases, West-German Centre for Infectious Diseases, University Duisburg-Essen, University Hospital Essen, Essen, North Rhine-Westphalia Germany; 4 Institute of Medical Immunology, Charité–Universitätsmedizin Berlin, Corporate Member of Freie Universität Berlin, Humboldt-Universität Zu Berlin, and Berlin Institute of Health, Berlin, Germany; 5 Department of Anaesthesiology and Intensive Care Medicine, West-German Centre for Infectious Diseases, University Duisburg-Essen, University Hospital Essen, Essen, North Rhine-Westphalia Germany; 6 Department of Gynaecology and Obstetrics, Ruhr-University Bochum, Marien Hospital Herne, University Hospital of the Ruhr-University Bochum, Herne, North Rhine-Westphalia Germany; 7 Department of Immunology, Leibniz Research Centre for Working Environment and Human Factors at the Technical University Dortmund (IfADo), Dortmund, North Rhine-Westphalia Germany; 8 Department of Molecular and Medical Virology, Ruhr-University Bochum, Bochum, North Rhine-Westphalia Germany; 9 Institute for Virology, University Duisburg-Essen, University Hospital Essen, Essen, North Rhine-Westphalia Germany

Patients with low renal function have an increased risk of critical COVID-19. While chronic kidney disease is associated with impairment of the immune system, it is still not known whether their worse COVID-19 outcome can be explained by a weaker antiviral response or by systemic inflammation. Importantly, there is still no literature on the immunological characteristics of COVID-19 patients with low renal function, which could potentially explain their increased fatality rate. Here, we performed an observational cohort study on 173 consecutively enrolled hospitalized COVID-19 patients, a non-vaccinated cohort recruited in 2020, which was classified according to their estimated glomerular filtration rate (eGFR) at admission. We analysed the immunological differences between patients with low and normal renal function, including circulating T and B cell subsets, SARS-CoV-2 reactive T cells and serum cytokines in follow-up. For more details on the methods, see the Supplementary Methods.

The patients were recruited (initial visit) at a median of 3 [IQR 1–6] days after the first positive PCR test. One hundred forty-three (82.7%) patients showed normal renal function at admission (hereafter Normal-eGFR; eGFR > 60 ml/min/1.73m^2^), while 30 patients (17.3%) suffered from low renal function (Low-eGFR; < 60 ml/min/1.73m^2^). Low-eGFR patients had significantly higher age (*P* < 0.001; see Table S1) and Charlson comorbidity index (*P* = 0.002). Therefore, we controlled for these factors in our analyses employing multivariate regression, as explained in the Supplementary Methods. Twelve patients (7.3%) died during follow-up; the Normal-eGFR sub-cohort had 5 fatal cases (3.5%), while for Low-eGFR there were 7 (23.3%). The association between Low-eGFR and patient death was significant (*P* = 0.022), independently from confounders.

We first analysed general immunological parameters: Almost all patients were lymphopenic (Fig. 1a). We observed significantly higher neutrophil counts among patients in the Low-eGFR group (Fig. 1b). Higher neutrophil counts have been associated with increased COVID-19 severity [1]. Regarding the T cell subset, patients with low eGFR had higher levels of in vivo activated HLA-DR^+^ CD4^+^ and CD8^+^ T cells, albeit without reaching statistical significance (Figure S1). On the other hand, we found significantly increased levels for terminally differentiated CD11a^++^CD28^−^CD57^+^ T cells among Low-eGFR at follow-up (Fig. 1c, d). These cells express markers of tissue migration (CD11a^++^) and do not require costimulatory molecules for activation (CD28^−^). This is in line with previous studies that identified such alterations in COVID-19 critical versus less severe disease manifestations [2]. Furthermore, an increase in CD28^−^CD57^+^ T cells has been observed repeatedly in a context of severe CKD [3]. These cells also expressed high levels of CD11a, a marker highly expressed in memory T cells and associated with activation and tissue migration [2]. CD11a^++^CD28^−^CD57^+^CD8^+^ T cells are associated with bystander activation in inflamed tissue, significantly contributing to tissue damage [4]. Higher frequencies of this cell subset might therefore contribute to the worse clinical outcome of patients with lower eGFR. Since these terminally differentiated cells are likely associated with ageing and chronic antigen exposure, we analysed the T cell memory composition in the patient cohorts but did not find any significant differences between the sub-cohorts (Figure S2). Finally, we did not find any significant differences between the study sub-cohorts for the SARS-CoV-2-specific T cell response (Fig. 1e, f) nor in the B cell compartment (Figure S3).

**Fig. 1:**
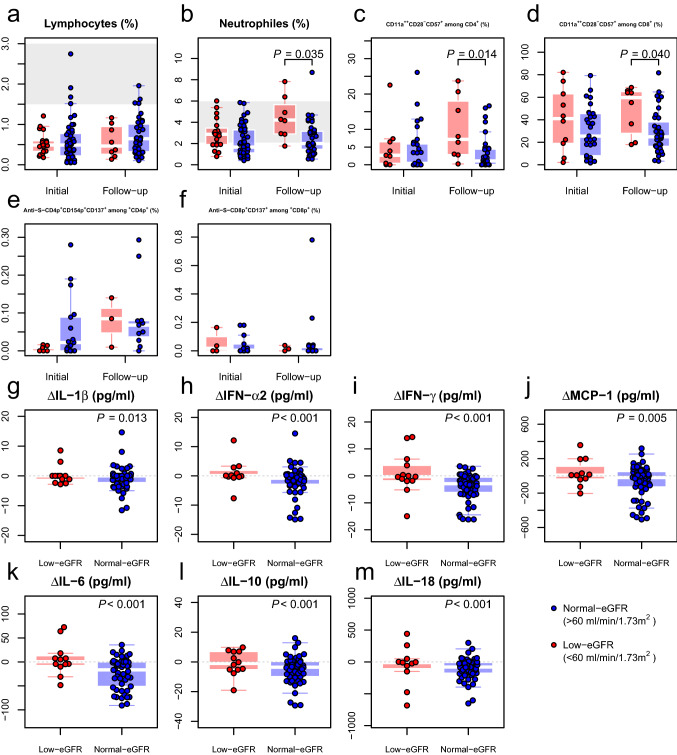
Patients in the Low-eGFR sub-cohort demonstrated an altered immune system. The figure depicts the levels of circulating immune subsets at the initial and follow-up visit (**a**–**f**), as well as the changes in serum cytokines between the two visits (**g**–**m**). For the immune subsets, peripheral blood from 57 patients, 41 from the Normal-eGFR sub-cohort (blue) and 16 from the Low-eGFR sub-cohort (red) was characterized. The figure depicts the frequencies of circulating lymphocytes (**a**), neutrophils (**b**), terminally differentiated T cells (**c**, **d**) and T cells specific against the viral spike antigen (**e**–**f**) using multiparametric flow cytometry. The P values were calculated controlling for differences in age and Charlson comorbidity index (see Statistical methods). In all cases, the left boxplots show the data for the initial visit, while the right boxplots depict the data at follow-up. The area shaded in grey represents the reference range for each parameter, if applicable. For the cytokine kinetics (**g**–**m**), 74 patients were analysed, 61 Low-eGFR sub-cohort (red) and 13 Normal-eGFR sub-cohort (blue). The figure shows the change in cytokine levels between initial and follow-up for each patient, where a negative value means a decrease in concentration and vice versa. A dashed grey line marks the threshold of a null change between the visits. Importantly, the P value does not refer to a comparison between the two sub-cohorts, but to the significance of the change in cytokine concentrations within each sub-cohort. *eGFR* estimated glomerular filtration rate; *IFN* interferon; *IL* interleukin; *MCP* monocyte chemoattractant protein

We further examined the differences in the cytokine profiles of the two sub-cohorts. While there were no significant differences in cytokine concentrations at either study visit (Figure S4), there was a significant decrease in the concentration of IL-1β, IFN-α2, IFN-γ, MCP-1, IL-6, IL-10 and IL-18 in the Normal-eGFR sub-cohort during follow-up, while the cytokine level remained unchanged in the Low-eGFR sub-cohort (Fig. 1g–m). We hypothesize that the latter dynamics are the result of chronic systemic inflammation. Previous studies on patients with senescent immunity demonstrate that chronic inflammation is associated with a higher mortality through infection [5]. Therefore, cytokine data suggest that patients with impaired kidney function might suffer from chronic inflammation, which could cause higher immunopathology and overall mortality.

In summary, we present a characterization of the immune system of COVID-19 patients with reduced renal function. Here, low eGFR emerges as a factor associated with T cell immunosenescence and an altered inflammatory response. These immunological alterations could potentially explain the worse disease outcomes of patients with reduced renal function. Main limitations of our work include the low number of patients within the Low-eGFR group, availability of sufficient samples, the differences in age and co-morbidities between the sub-cohorts and the fact that the cohort was recruited between spring and autumn 2020. Further, prospective studies with larger patient cohorts and long-term follow-up data are needed to confirm our observations.

COVID-19, chronic kidney disease, renal function, immune system, immunosenescence.

## Supplementary Information

Below is the link to the electronic supplementary material.

Supplementary methods (DOCX 31 kb)

Supplementary Figure 1 (PDF 15 kb)

Supplementary Figure 2 (PDF 44 kb)

Supplementary Figure 3 (PDF 31 kb)

Supplementary Figure 4 (PDF 288 kb)

Supplementary Table 1 (DOCX 16 kb)

## Data Availability

Limited data access requests can be sent to Nina Babel (nina.babel@charite.de).
