# Supplementary material for: Impact of low eGFR on the immune response against COVID-19
Source: J Nephrol. 2022 Jul 2;36(1):199–202. doi: 10.1007/s40620-022-01374-1 (PMC9895010; doi:10.1007/s40620-022-01374-1)
Supplement: Supplementary file 1 — Supplementary methods (DOCX 31 kb) [file 40620_2022_1374_MOESM1_ESM.docx]

# Supplementary Methods

## Study population and design

173 consecutive COVID-19 patients from the University Hospitals Bochum and Essen (North Rhine-Westphalia, Germany) were recruited between March and October 2020. Inclusion criteria comprised a positive SARS-CoV-2 PCR test and available eGFR data at recruitment; exclusion criteria were an admission to ICU over seven days before recruiting and the usage of haemodialysis prior to admission. The patients were recruited (initial visit) at a median of 3 [IQR 1-6] days after the first positive PCR test. Based on the eGFR at admission, the patients were classified into two sub-cohorts: Low-eGFR and Normal-eGFR, employing a threshold of 60 ml/min/1.73m^2^. This threshold was chosen because it is the reference for the definition of CKD in the KDIGO guideline and an eGFR below 60 ml/min/1.73m^2^ is associated in the general population with higher mortality risk.[11,20,21] This threshold has been employed repeatedly to analyse the impact of eGFR on COVID-19 outcomes and vaccination response.[16,19,22–24] Demographic and clinical characteristics of the sub-cohorts are shown in Table S1.

## Definition of study visits

The eGFR was monitored along a maximum of seven visits during hospitalization, calculated employing the CKD-EPI formula.[25] These visits were scheduled at study admission and 3, 7, 11, 15, 22, and 29 days after admission. Furthermore, the cellular immune response and the cytokine serum levels of the patients were monitored at the initial visit and at a second time point (follow-up visit). A follow-up visit on the other hand was available for 88 (50.9%).

## Characterization of the cellular immune system

The cellular immune response was analysed employing flow cytometry data, as described before.[1,2] Shortly, granulocytes, monocytes and lymphocytes were distinguished by their CD45 expression and side scatter profile. Granulocytes were separated by CD16 in eosinophils (CD16^-^) and neutrophils (CD16^+^). Lymphocytes were separated in NK (CD3^-^CD56^+^) cells, T cells (CD3^+^CD56^–^) and B cells (CD19^+^). T cell populations were defined as CD3^+^CD4^+^ or CD3^+^CD8^+^ and were phenotyped employing surface markers HLA-DR, CD11a, CD28, CD57, CD45RA, CCR7; CD45RA^-^CCR7^+^ were defined as central memory T cells, CD45RA^+^CCR7^-^ as TEMRA cells, CD45RA^-^CCR7^-^ as effector memory and CD45RA^+^CCR7^+^ as naive T cells. B cells were identified by the expression of CD19 and separated into naive (IgD^+^CD27^-^) and marginal zone B cells (IgD^+^CD27^+^). Additionally, B cells were separated by expression of IgM and IgD into double negative (IgM^-^IgD^-^) and double positive (IgM^+^IgD^+^) cells. Double negative cells could be separated by CD38 and CD27 into switched memory B cells (CD27^-/+^CD38^-/+^) and plasmablasts (CD27^++^CD38^++^). IgM^+^IgD^+^CD38^++^CD24^++^ cells were classified as transitional B cells.

The analysis of virus-specific T cells was performed by stimulation using overlapping peptide pools of the wild type SARS-CoV-2 spike (S) protein, as reported before.[1,2] Shortly, the peptide pool were dissolved at a concentration of 1 mg/mL. 2.5 ·10^6^ peripheral blood mononuclear cells were thawed and plated and were stimulated for 16 h. As a positive control we employed staphylococcal enterotoxin B, the negative control was with vehicle. Antiviral response was defined employing the surface markers CD154 and CD137: CD154^+^CD137^+^CD4^+^ T cells and CD137^+^CD8^+^ were therefore considered virus-specific. We considered as detectable a virus-specific response of over 0.01% after subtracting the negative control background. All values below 0.01% were set to zero.

Flow cytometry data were analysed using FlowJo version 10.6.2 (BD Biosciences).

## Measurement of serum cytokine profile

The LEGENDplex Human Inflammation Panel 1 (13-plex) (BioLegend, CA, USA) containing interleukin (IL)-1β, interferon (IFN)-α2, IFN-γ, tumor necrosis factor (TNF)-α, monocyte chemoattractant protein (MCP)-1 (CCL2), IL-6, IL-8, IL-10, IL-12p70, IL-17A, IL-18, IL-23, and IL-33 was used according to the manufacturer’s instruction. The concentration of the analytes was calculated using the LEGENDplex Data Analysis Software v.2020.12.15.

## Statistical analysis

Statistical analysis was performed using R, version 3.6.2.[5] Categorical variables are reported as numbers and frequencies; quantitative variables are reported as median and interquartile range. No imputation of missing data was employed. The applied statistical tests are two-sided. Changes within a variable are tested by the one-sample Wilcoxon signed rank test. Correlations were determined employing the Spearman coefficient. The independence of the effect of eGFR on outcome from confounders was assessed by logistic regression, with the outcome as dependent variable and the eGFR sub-cohort and confounders as independent values. The P values for the regression models were calculated employing the *t*-test.

In the evaluation of the two sub-cohorts, we controlled for the potential effects of age and Charlson comorbidity index by multivariate regression. For all parameters with a significant or borderline significant difference between the two sub-cohorts (P<0.100), a multivariate linear regression analysis was performed. The reported P value in the figures corresponds to the P value of the sub-cohort in the multivariate analysis.

For depiction purposes, extremely high values of variables were excluded employing Tukey’s fences (k=3), estimated for all values over the detection limit. P values below 0.050 were considered significant; only significant P values are reported in the figures. P values were not corrected for multiple testing, as this study was of exploratory nature.[8]

1. Thieme CJ, Anft M, Paniskaki K, Blazquez-Navarro A, Doevelaar A, Seibert FS, Hoelzer B, Konik MJ, Berger MM, Brenner T, Tempfer C, Watzl C, Meister TL, Pfaender S, Steinmann E, Dolff S, Dittmer U, Westhoff TH, Witzke O, Stervbo U, Roch T, Babel N. Robust T Cell Response Toward Spike, Membrane, and Nucleocapsid SARS-CoV-2 Proteins Is Not Associated with Recovery in Critical COVID-19 Patients. *Cell Reports Med*. 2020;1(6):100092. doi:10.1016/j.xcrm.2020.100092

2. Anft M, Paniskaki K, Blazquez-Navarro A, Doevelaar A, Seibert FS, Hölzer B, Skrzypczyk S, Kohut E, Kurek J, Zapka J, Wehler P, Kaliszczyk S, Bajda S, Thieme CJ, Roch T, Konik MJ, Berger MM, Brenner T, Kölsch U, Meister TL, Pfaender S, Steinmann E, Tempfer C, Watzl C, Dolff S, Dittmer U, Abou-El-Enein M, Westhoff TH, Witzke O, Stervbo U, Babel N. COVID-19-Induced ARDS Is Associated with Decreased Frequency of Activated Memory/Effector T Cells Expressing CD11a++. *Mol Ther*. 2020;28(12):2691-2702. doi:10.1016/j.ymthe.2020.10.001

3. Bajda S, Blazquez-Navarro A, Samans B, Wehler P, Kaliszczyk S, Amini L, Schmueck-Henneresse M, Witzke O, Dittmer U, Westhoff TH, Viebahn R, Reinke P, Thomusch O, Hugo C, Olek S, Roch T, Babel N. The role of soluble mediators in the clinical course of EBV infection and B cell homeostasis after kidney transplantation. *Sci Rep*. 2020;10(1):1-13. doi:10.1038/s41598-020-76607-z

4. Stervbo U, Bajda S, Wehler P, Rohn BJ, Streichhahn M, Temizsoy S, Kohut E, Roch T, Viebahn R, Westhoff TH, Babel N. Stability of 12 T-helper cell-associated cytokines in human serum under different pre-analytical conditions. *Cytokine*. 2020;129:155044. doi:10.1016/j.cyto.2020.155044

5. R Core Team, R Development Core Team. R: A language and environment for statistical computing. 2020. http://www.r-project.org/.

6. Jennrich RI. An Asymptotic χ^2 Test for the Equality of Two Correlation Matrices. *J Am Stat Assoc*. 1970;65:904-912.

7. Wei T, Simko V. R package ‘corrplot’: Visualization of a Correlation Matrix. 2017. https://github.com/taiyun/corrplot.

8. Bender R, Lange S. Adjusting for multiple testing - when and how? *J Clin Epidemiol*. 2001;54:343-349. doi:10.1016/S0895-4356(00)00314-0
