# Supplementary material for: Impact of low eGFR on the immune response against COVID-19
Source: J Nephrol. 2022 Jul 2;36(1):199–202. doi: 10.1007/s40620-022-01374-1 (PMC9895010; doi:10.1007/s40620-022-01374-1)
Supplement: Supplementary file 2 — Supplementary Figure 1 (PDF 15 kb) [file 40620_2022_1374_MOESM2_ESM.pdf]

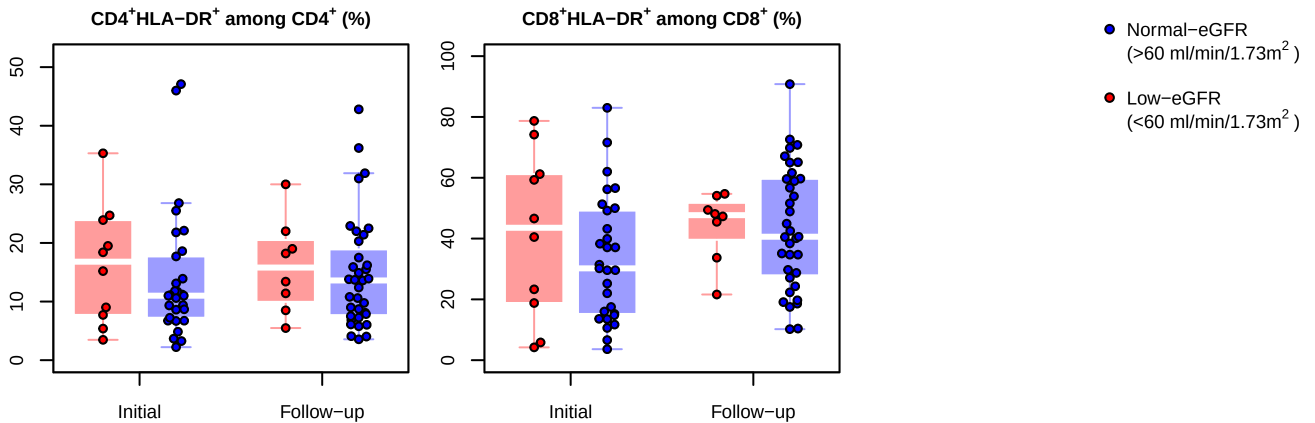

CD4+HLA−DR+ among CD4+ (%)
CD8+HLA−DR+ among CD8+ (%)
0
10
20
30
40
50
0
20
40
60
80
100
Initial
Follow−up
Initial
Follow−up
Normal−eGFR
(>60 ml/min/1.73m2)
Low−eGFR
(<60 ml/min/1.73m2)
