# Supplementary material for: Impact of low eGFR on the immune response against COVID-19
Source: J Nephrol. 2022 Jul 2;36(1):199–202. doi: 10.1007/s40620-022-01374-1 (PMC9895010; doi:10.1007/s40620-022-01374-1)
Supplement: Supplementary file 3 — Supplementary Figure 2 (PDF 44 kb) [file 40620_2022_1374_MOESM3_ESM.pdf]

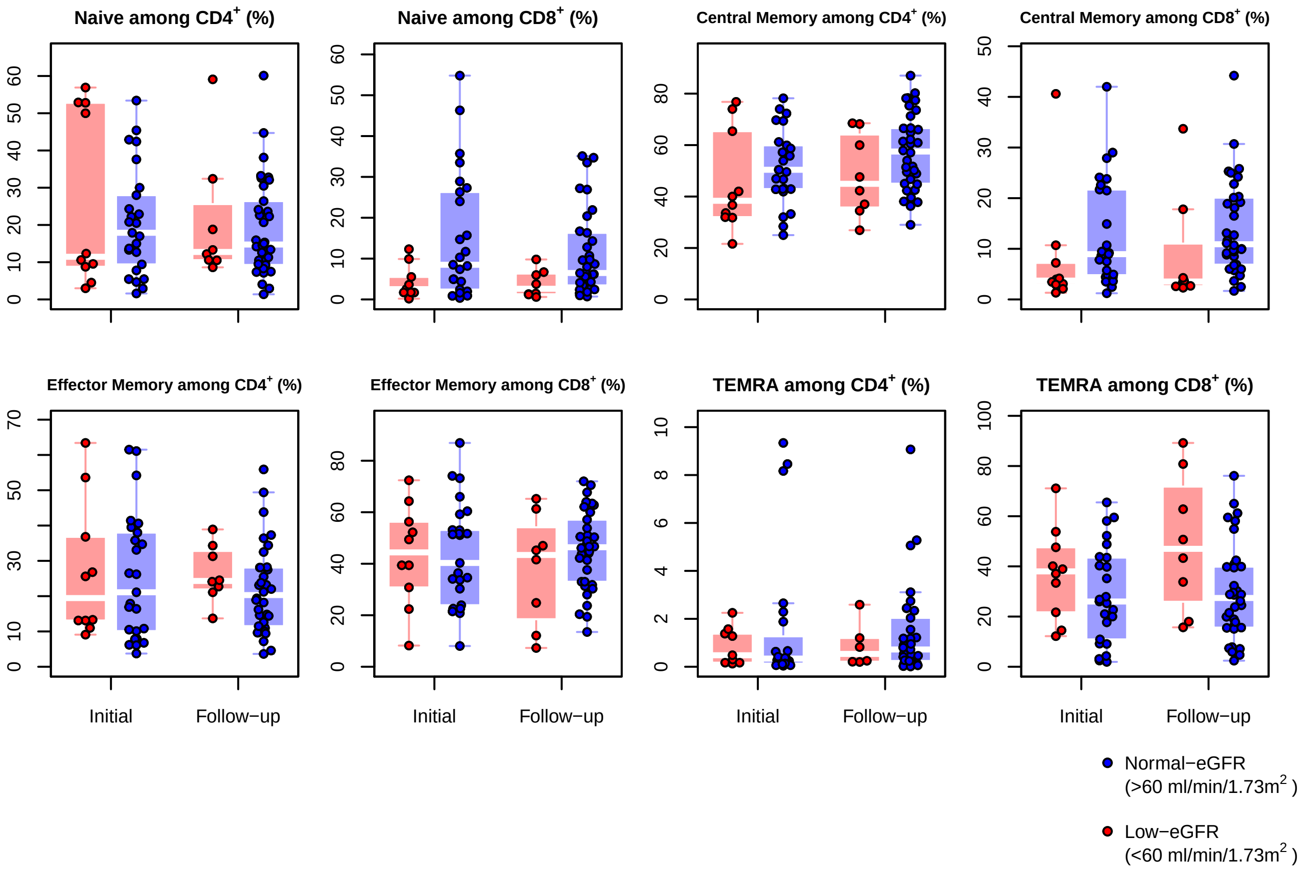
Naive among CD4+ (%)
Naive among CD8+ (%)
Central Memory among CD4+ (%)
Central Memory among CD8+ (%)
Effector Memory among CD4+ (%)
Effector Memory among CD8+ (%)
TEMRA among CD4+ (%)
TEMRA among CD8+ (%)
Initial
Follow−up
Normal−eGFR (>60 ml/min/1.73m2)
Low−eGFR (<60 ml/min/1.73m2)
