# Supplementary material for: Impact of low eGFR on the immune response against COVID-19
Source: J Nephrol. 2022 Jul 2;36(1):199–202. doi: 10.1007/s40620-022-01374-1 (PMC9895010; doi:10.1007/s40620-022-01374-1)
Supplement: Supplementary file 4 — Supplementary Figure 3 (PDF 31 kb) [file 40620_2022_1374_MOESM4_ESM.pdf]

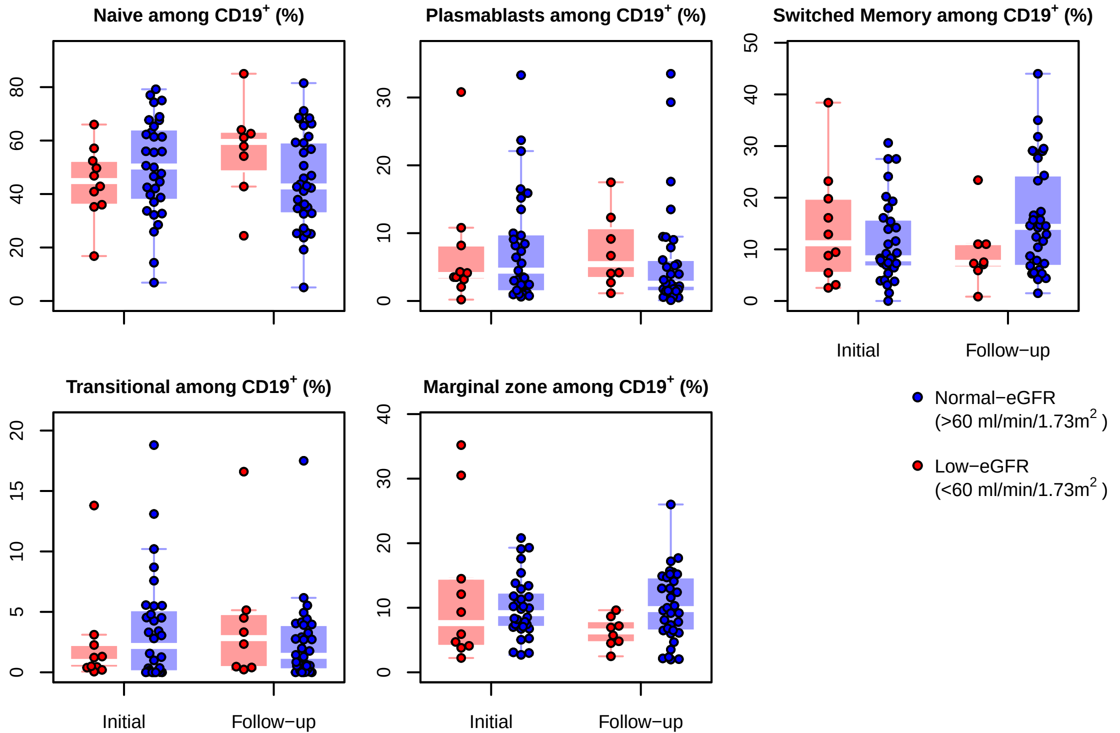
Naive among CD19+ (%)
Plasmablasts among CD19+ (%)
Switched Memory among CD19+ (%)
Transitional among CD19+ (%)
Marginal zone among CD19+ (%)
0
20
40
60
80
0
10
20
30
0
10
20
30
40
50
0
5
10
15
20
0
10
20
30
40
Initial
Follow−up
Normal−eGFR
(>60 ml/min/1.73m2)
Low−eGFR
(<60 ml/min/1.73m2)
