# Supplementary figures and images for: Impact of low eGFR on the immune response against COVID-19
Source: J Nephrol. 2022 Jul 2;36(1):199–202. doi: 10.1007/s40620-022-01374-1 (PMC9895010; doi:10.1007/s40620-022-01374-1)

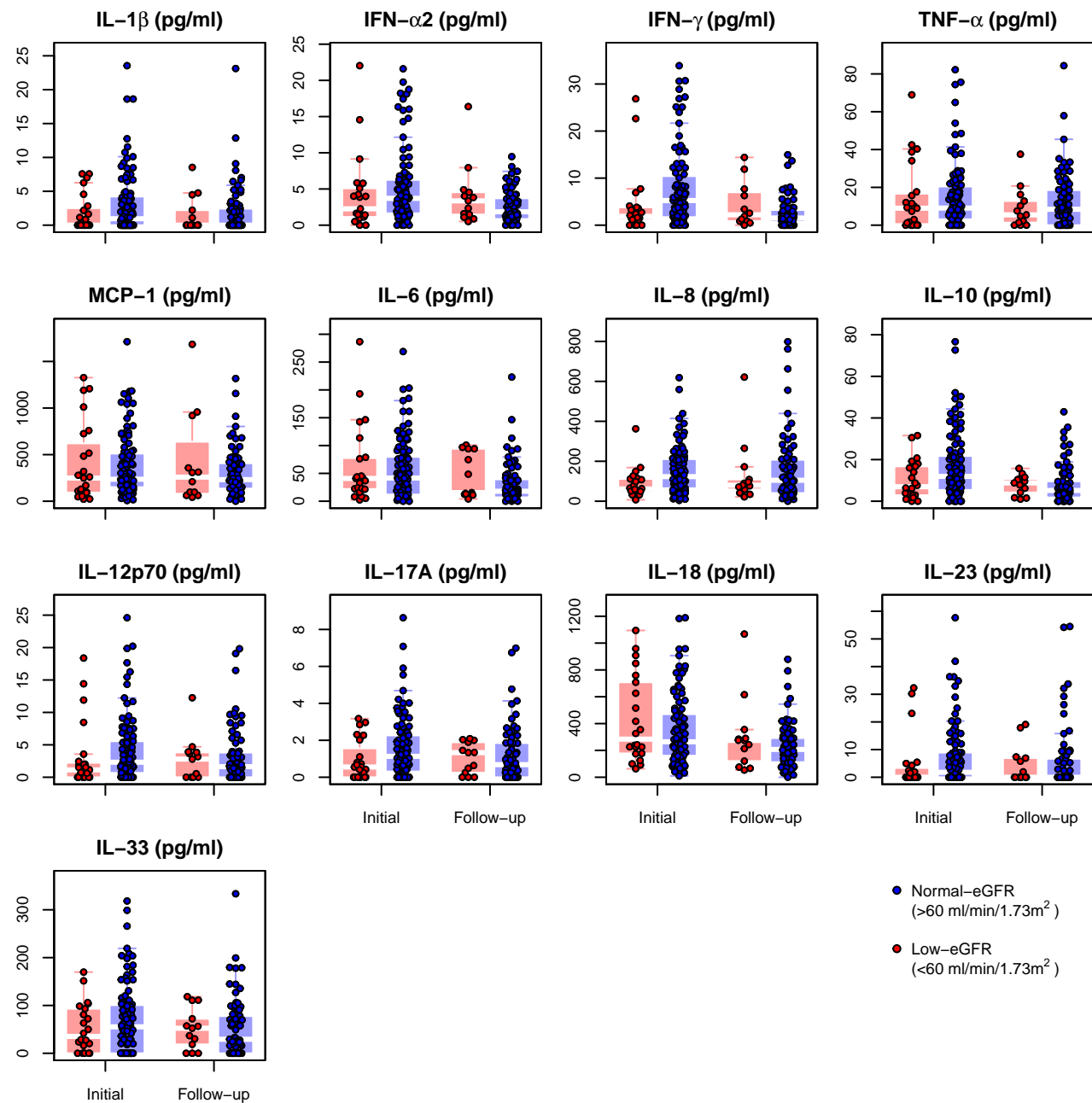

Supplement: Supplementary file 5 — Supplementary Figure 4 (PDF 288 kb) [file 40620_2022_1374_MOESM5_ESM.pdf]
