# Supplementary material for: Impact of low eGFR on the immune response against COVID-19
Source: J Nephrol. 2022 Jul 2;36(1):199–202. doi: 10.1007/s40620-022-01374-1 (PMC9895010; doi:10.1007/s40620-022-01374-1)
Supplement: Supplementary file 6 — Supplementary Table 1 (DOCX 16 kb) [file 40620_2022_1374_MOESM6_ESM.docx]

# Supplemental materials

**Supplementary methods**

**Table S1**. Demographic, monitoring and outcome information of the patient cohort as a function of their eGFR at enrolment, where Normal-eGFR is defined as those patients with an eGFR > 60 ml/min/1.73m^2^ and Low-eGFR had an eGFR below the threshold.

| **Variable** | **Low-eGFR (N=30)** | **Normal-eGFR**  **(N=143)** | **P value** |
| --- | --- | --- | --- |
| Male patients | 15 (50.0%) | 85 (59.4%) | 0.417 |
| Age at recruiting (years) | 76 [62-83] | 57 [42-69] | <0.001 |
| Charlson comorbidity index | 2 [0-4] | 0 [0-2] | 0.002 |
| History of CKD | 7 (23.3%) | 0 (0.0%) | <0.001 |
| Initial visit (days post-diagnose) | 3 [2-5] | 3 [1-6] | 0.563 |
| Follow-up visit (days post-diagnose) | 12 [5-22] | 9 [6-15] | 0.663 |

**Figure S1. No significant differences between sub-cohorts in the levels of HLA-DR^+^ T cells.** ﻿Peripheral blood from 53 patients, 39 from the Normal-eGFR sub-cohort (blue) and 14 from the Low-eGFR sub-cohort (red) was characterized for T cell subsets. The figure depicts the frequencies of the T cell activation HLA-DR using multiparametric flow cytometry. The P values were calculated controlling for differences in age and Charlson comorbidity index (see Statistical methods). In all cases, the left boxplots show the data for the initial visit, while the right boxplots depict the data at follow-up. eGFR: estimated glomerular filtration rate; HLA: human leucocyte antigen.

**Figure S2. No significant differences between sub-cohorts were observed in the T cell memory subsets.** ﻿Peripheral blood from 53 patients, 39 from the Normal-eGFR sub-cohort (blue) and 14 from the Low-eGFR sub-cohort (red) was characterized for T cell memory subsets. The P values were calculated controlling for differences in age and Charlson comorbidity index (see Statistical methods). In all cases, the left boxplots show the data for the initial visit, while the right boxplots depict the data at follow-up. eGFR: estimated glomerular filtration rate.

**Figure S3. No significant differences between sub-cohorts were observed in the B cell subsets.** ﻿Peripheral blood from 53 patients, 39 from the Normal-eGFR sub-cohort (blue) and 14 from the Low-eGFR sub-cohort (red) was characterized for B cell subsets. The P values were calculated controlling for differences in age and Charlson comorbidity index (see Statistical methods). In all cases, the left boxplots show the data for the initial visit, while the right boxplots depict the data at follow-up. eGFR: estimated glomerular filtration rate.

**Figure S4. No significant differences between sub-cohorts in the cytokine levels.** ﻿Serum from 133 patients, 110 from the Normal-eGFR sub-cohort (blue) and 23 from the Low-eGFR sub-cohort (red) was characterized for 13 cytokines employing a multiplex cytokine assay. The P values were calculated controlling for differences in age and Charlson comorbidity index. In all cases, the left boxplots show the data for the initial visit, while the right boxplots depict the data at follow-up. eGFR: estimated glomerular filtration rate; IFN: interferon; IL: interleukin; MCP: monocyte chemoattractant protein; TNF: tumor necrosis factor.
